# Memory-phenotype CD4^+^ T Lymphocytes: A Novel Therapeutic Target in Infectious or Autoimmune Diseases?

**DOI:** 10.31662/jmaj.2022-0048

**Published:** 2022-06-17

**Authors:** Takeshi Kawabe

**Affiliations:** 1 Department of Microbiology and Immunology, Tohoku University Graduate School of Medicine, Sendai, Japan

**Keywords:** CD4^+^ T lymphocytes, Homeostasis, Innate immunity, Autoimmunity

## Abstract

Infectious diseases are posing threats to the world. Although several types of antibiotics and antivirals have been created to treat the diseases, emerging/re-emerging infectious diseases, as well as those caused by pathogens with multidrug resistance, remain to be significant challenges. As a new therapeutic approach, “host-directed therapy” that enhances immune responses of host cells has been proposed. Nevertheless, the agents used in this strategy often lead to a side effect of hyperinflammation, posing a challenge in developing safe and effective drugs. In this review, I suggest boosting a novel CD4^+^ T lymphocyte population called “memory-phenotype (MP) cells” as a target of the host-directed therapy. MP cells are homeostatically generated from peripheral naïve precursors via recognition of self rather than foreign antigens and are maintained via rapid proliferation in steady state. Surprisingly, MP cells possess innate immune function; they can respond to an inflammatory cytokine IL-12 in the absence of antigen recognition to produce IFN-γ, thereby contributing to host defense against *Toxoplasma* and *Mycobacterium*. In this article, I summarize our current understanding of the mechanisms of generation, maintenance, differentiation, and innate effector function of MP CD4^+^ T lymphocytes and further discuss how we can target these cells as a new therapeutic strategy to infectious and autoimmune/inflammatory diseases.

## Introduction

Infectious diseases have been threatening all countries. As conventional therapeutic strategies to these diseases, various types of antimicrobials including antibiotics and antivirals have been developed, and such efforts have thus far contributed to a significant reduction in disease incidence ^(1), (2)^. However, because each of these drugs generally has a limited range of pathogen targets, we are faced with difficulties in developing effective agents against emerging and re-emerging infectious diseases including coronavirus disease 2019 and those induced by pathogens with multidrug resistance.

Besides the conventional antimicrobial therapies, a new approach called “host-directed therapy” has been proposed ^(3)^. Host-directed therapy is aimed at curing and/or ameliorating infectious diseases by enhancing the immune responses of the host or by interfering with host cell mechanisms that are required for pathogen replication/persistence. For example, type I IFN has been used for the treatment of patients with hepatitis B or C ^(4)^ whereas IFN-γ may improve lung repair in *Mycobacterium tuberculosis* infection and reduce infection-related deaths in trauma patients with sepsis^(5), (6), (7), (8)^. However, a major problem of these therapies is their side effect; they can induce inflammation-related symptoms such as fever, fatigue, and autoimmunity.

The immune system comprises two parts: innate and adaptive immunity, and in the latter response, CD4^+^ T lymphocytes play an essential role. Because of their critical function, dysfunction of T cells is well known to cause immunodeficiency while their excess activation can lead to the onset of autoimmune and inflammatory diseases ^(9), (10), (11)^. Among CD4^+^ T cells, we have recently reported a novel cell subpopulation that spontaneously arises from naïve precursors under homeostatic conditions in mice ^(12), (13)^. This cell population is very similar to conventional, foreign antigen (Ag)-specific memory CD4^+^ T lymphocytes in their phenotype; surprisingly, however, these “memory-phenotype (MP)” CD4^+^ T cells can exert innate immune function. Hence, they respond to an inflammatory cytokine IL-12 in the absence of Ag recognition and produce IFN-γ, thereby contributing to host defense against *Toxoplasma gondii* and *M. tuberculosis*. Moreover, this innate IFN-γ production has been shown to be critical for the later development of Ag-specific T cell immune responses, highlighting MP cells as a novel T lymphocyte population bridging innate and adaptive immunity ^(14), (15)^.

Based on the above innate feature of MP CD4^+^ T lymphocytes, it could be a good strategy to deliberately boost their immune activity while carefully inhibiting excess inflammation in host-directed therapy against infectious diseases. In this article, I will review our current understanding of the development, maintenance, and differentiation of MP CD4^+^ T cells as well as their innate effector function in host defense, and further discuss these cells as a potential therapeutic target in infectious and autoimmune/inflammatory diseases.

## “Rediscovery” of MP CD4^+^ T Lymphocytes

CD4^+^ T lymphocytes play an essential role in adaptive immune responses. Thus, they distinguish pathogen-derived foreign Ags from self and only attack the former elements. T lymphocytes are originally generated in the thymus where they are tested for their self-reactivity through positive and negative selection ^(16)^. Specifically, thymocytes expressing T cell receptors (TCRs) that strongly bind to self-Ags are signaled to undergo apoptosis while those that fail to react with self-peptide/major histocompatibility complex (MHC) molecules are deleted by “death by neglect.” Consequently, only thymocytes that have TCRs with low affinity to self-Ag/MHC complexes survive and migrate into the periphery as T lymphocytes.

In the periphery, CD4^+^ T lymphocytes are typically divided into three subpopulations: naïve, effector, and memory cells ^(11)^. In infectious conditions, naïve CD4^+^ T cells that have TCRs specific for the pathogen Ags bound to self-MHC extensively proliferate to differentiate into effector cells to contribute to host protection. After the pathogen is cleared, most effector T cells are deleted via apoptosis, leaving a small population of memory cells that provide the hosts with immunological memory. These CD4^+^ T cell populations are strictly regulated by homeostatic mechanisms, and their disruption can lead to immunodeficiency or autoimmune/inflammatory diseases as described above.

In unimmunized mice where overt immune responses toward foreign Ags are absent, conventional CD4^+^ T lymphocytes comprise naïve (CD44^lo^ CD62L^hi^) and memory (CD44^hi^ CD62L^lo^) compartments ^(11)^. Because the latter population adopts a phenotype that is shared by conventional, foreign Ag-specific memory/effector cells, it was presumed that this population all represents conventional memory cells that are specific for some foreign Ags derived from commensal microbes and/or food.

Nevertheless, this assumption has been now questioned for several reasons. First, CD44^hi^ CD62L^lo^ CD4^+^ T lymphocytes can be generated in the absence of foreign Ags. Thus, the same cell population has been shown to be present not only in specific pathogen-free (SPF) but also in germ-free (GF) and even in antigen-free (AF) environments^(12), (17), (18), (19)^. Because AF mice are deprived of both commensal and food Ags, self-Ags are thought to be the major stimulus for the development of CD44^hi^ CD4^+^ T cells present in these animals. Similarly, we have recently shown that CD44^hi^ CD4^+^ T cells are present in TCR-transgenic mice where all T lymphocytes possess a single TCR specific for a defined foreign Ag ^(13)^. Since these animals were not given the cognate foreign Ags in the same study, this observation strongly supports the notion that self-Ags are sufficient for the generation of CD44^hi^ CD4^+^ T lymphocytes at homeostasis.

The second reason is that CD44^hi^ CD4^+^ T cells rapidly proliferate in steady state, whereas foreign Ag-specific memory cells are generally quiescent. Specifically, ~10% of CD44^hi^ CD4^+^ T lymphocytes undergo proliferation in one day under homeostatic conditions while only ~2% of Ag-specific memory cells divide in the same period ^(20), (21)^, and at any given time point at homeostasis, ~70% have proliferated at least once over the past 4 weeks ^(22)^. These observations suggest that CD44^hi^ CD4^+^ T lymphocytes comprise a major cell population that is dynamically maintained by rapid proliferation.

The third reason lies in the finding that CD44^hi^ CD4^+^ T lymphocytes are unique in their differentiation pathways. In the case of conventional helper T cell differentiation, upon foreign Ag recognition, naïve cells differentiate into distinct subsets including Th1, Th2, and Th17 ^(23)^. These subsets express master transcription factors T-bet, Gata3, and RORγt, respectively, and exert unique effector functions by producing different types of cytokines. This fate decision is mainly determined by environmental cytokines. For example, Th1 differentiation is driven by IL-12, whereas IL-4 plays an important role in Th2 development ^(24), (25), (26)^. The former cytokine is produced by dendritic cells (DCs) upon ligation of foreign agonists including LPS ^(27), (28)^. Conversely, steady state CD44^hi^ CD4^+^ T lymphocytes have been recently shown to include a T-bet-expressing Th1-like subset at homeostasis; nevertheless, its differentiation seems to be self-driven rather than foreign agonist-dependent ^(13)^. Given that AF mice have unaltered levels of T-bet^+^ CD44^hi^ CD4^+^ T cells ^(13)^, their differentiation is supposed to be self-sufficient as opposed to conventional helper T cell differentiation where foreign agonists play a dominant role.

Fourth, in the case of CD8^+^ T lymphocytes, steady state CD44^hi^ and conventional memory cells are known to be distinguishable from each other using a marker. Specifically, the former cells are integrin α_4_^lo^, whereas the latter ones are α_4_^hi^ ^(29)^. Although the same marker does not seem to be applicable for CD4^+^ T cells ^(14), (30)^, some marker(s) can likely distinguish naturally arising CD44^hi^ CD4^+^ T lymphocytes from foreign Ag-specific memory cells.

Based on the above considerations, CD44^hi^ CD62L^lo^ CD4^+^ T lymphocytes present in SPF, GF, and AF animals are now thought to represent a cell population that is different from conventional, foreign Ag-specific memory cells. We have been referring to this self-driven population as “MP cells” ^(11), (14), (15)^. Interestingly, an analogous CD4^+^ T cell population is also existent in human cord blood as well as fatal lymphoid organs, in the form of CD45RA^lo^ CD45RO^hi^ cells ^(31), (32)^. Because in these tissues, exposure to foreign Ags and agonists is very limited, self-Ags are considered to be the dominant force driving this CD4^+^ T lymphocyte subpopulation also in humans.

## The Origin of MP Cells

Because essentially all conventional CD4^+^ T lymphocytes newly generated from the thymus are naïve ^(12)^, MP cells are believed to derive from naïve precursors in the periphery. A clue to the question of how these cells are generated was first obtained from early studies where CD4^+^ T cells were transferred into unimmunized recipient mice that had been artificially made lymphopenic ^(33), (34)^. Thus, in these lymphopenic hosts such as irradiated, anti-Thy1 monoclonal antibody-treated, and *Rag1/2*-deficient mice, transferred donor cells spontaneously proliferated to acquire a CD44^hi^ CD62L^lo^ memory phenotype. Although in these initial studies, transferred CD4^+^ donor T lymphocytes included both naïve and MP cells, subsequent reports found purified naïve cells to have the same proliferative capacity ^(35), (36)^. This type of proliferative response that occurs in the absence of explicit challenge with foreign Ags is called “homeostatic proliferation.”

In lymphopenic settings, naïve CD4^+^ T lymphocytes show two types of cell division: “slow” and “fast” ^(35), (36)^. Slow proliferation is defined as a cell division of 2-3 times a week. Because resultant cells generated in this manner maintain a CD44^lo^ CD62L^hi^ naïve phenotype ^(36)^, this proliferation is thought to serve as a mechanism for reproducing naïve CD4^+^ T cells. This proliferation is dependent on self-Ag-recognition as well as homeostatic cytokine IL-7 ^(33), (35), (37), (38), (39)^. By contrast, some naïve CD4^+^ T cells proliferate more than once a day and spontaneously acquire a CD44^hi^ CD62L^lo^ memory phenotype via fast cell division ^(36)^. This form of proliferation is dependent on host-derived MHC molecules^(33), (37), (38), (40)^, demonstrating an essential role for Ag recognition in the response generation. Foreign and self-Ags seem to work as the stimuli for this proliferation. Thus, rapidly proliferating donor cells seen in an SPF environment are partially and significantly diminished in GF mice; interestingly, however, the fast cell division does not completely disappear in the latter animals ^(35), (41)^. It is unlikely that food Ags play a role in this residual response because fast-dividing cells are also existent in AF mice ^(39)^. These findings argue that both commensal and self-Ags can contribute to the induction of fast proliferation in lymphopenic conditions. This notion is further supported by the finding that commensal-dependent and commensal-independent fast-dividing cells can be distinguished by a marker α_4_β_7_ on donor T lymphocytes ^(42), (43)^.

**Figure 1.**
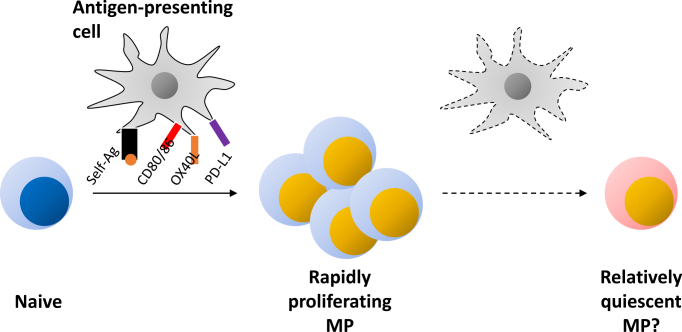
Steady state generation and maintenance of MP CD4^+^ T lymphocytes. A naïve CD4^+^ T lymphocyte recognizes both self-Ag/MHC complexes and costimulatory/coinhibitory molecules on antigen-presenting cells to convert to MP cells. Once generated, MP cells become less dependent on TCR signaling while retaining reactivity to costimulation/coinhibition for their tonic proliferation. Some of these rapidly dividing MP cells may gradually cease to proliferate with time.

Whether the above lymphopenia-induced homeostatic proliferation occurs in physiologic settings is an important question. In this regard, neonatal mice are well known to be lymphopenic and in such a situation naïve CD4^+^ T lymphocytes extensively proliferate to give rise to CD44^hi^ CD62L^lo^ cells via homeostatic expansion ^(44)^. Additionally, we have recently shown that naïve CD4^+^ T cells can spontaneously acquire a CD44^hi^ CD62L^lo^ memory phenotype even in adult, lymphosufficient settings through Ag recognition as well as CD28 costimulation (Figure 1) ^(12)^. Because this phenotypic conversion is equally present in SPF and GF environments, self rather than foreign Ags are thought to be the dominant driving force. We therefore propose self-driven homeostatic proliferation as a physiologic mechanism that is essential for the generation of MP CD4^+^ T lymphocytes in both neonatal and adult environments.

Given that MP CD4^+^ T cells are generated from naïve precursors via self-Ag-recognition, it is inferred that the former T cells have higher TCR affinity to self-Ags than do their naïve counterparts. How then is autoimmunity as a result of such self-reactivity inhibited in normal, healthy conditions? This is at least in part achieved by regulatory T cells (Tregs) ^(45)^. Tregs can inhibit immune responses of neighboring T lymphocytes in both Ag-specific and Ag-unspecific fashions, and because of this function, acute depletion of Tregs leads to lethal autoimmunity even in the AF environment ^(39), (46), (47)^. This suggests a critical role for Tregs in the inhibition of auto-inflammation driven by self-reactive T lymphocytes in steady state.

Additionally, a T cell-intrinsic mechanism called “tuning” is known to regulate TCR signal strength ^(48)^. Thus, two opposing factors, excitatory and de-excitatory, modify TCR signal strength and determine the activation threshold for T cell activation. For example, CD5 inhibits TCR signaling pathway via SHP-1 ^(49), (50)^, whereas miR-181a increases TCR sensitivity ^(51)^ in CD4^+^ T cells. These positive and negative regulators are thought to cooperatively “fine-tune” the activation threshold for overt autoimmune T cell responses.

## Proliferation for Maintenance

In the case of foreign Ag-specific memory CD4^+^ T cell formation, once generated memory T lymphocytes become MHC-independent ^(52)^ and this provides the essential mechanism that enables the same cells to survive for a long term in the absence of cognate foreign Ags after pathogen elimination. In such a situation memory CD4^+^ T cells adopt a quiescent state and only minimally divide in response to homeostatic cytokines IL-7 and/or IL-15 ^(20), (53), (54)^.

As for MP cells, although these cells seem to become less dependent on TCR signaling after development, a substantial fraction of MP cells tonically proliferates for their maintenance at homeostasis ^(12), (21)^. Besides IL-7, several types of costimulatory signaling delivered by CD28, OX40, GITR, and 4-1BB ligation can positively regulate this cell division, whereas CTLA-4 and PD-1 signaling inhibits the response (Figure 1) ^(12), (21), (55), (56)^. Considering that the apoptotic rate is also high in MP cells ^(12)^, their homeostasis is likely to be maintained by a rapid turnover.

It remains unknown whether all MP CD4^+^ T lymphocytes exhibit a rapid turnover or whether a part of them do so under homeostatic conditions. In this regard, we recently showed that although newly generated MP cells are essentially all Ki67^+^, they gradually cease to proliferate over a 2 month period ^(12)^. Hence, it is reasonable to speculate that self-driven MP cells comprise a mixture of a newly generated, fast proliferating cell population together with a more quiescent subpopulation that was generated many weeks ago (Figure 1). If this hypothesis is the case, the conversion rate from cycling to more stable MP fractions may be determined by the balance between costimulatory and coinhibitory signals described above. Further investigation is needed to elucidate the mechanism of this MP conversion as well as its immunological significance.

## Homeostatic Differentiation and Its Immunological Function

**Figure 2.**
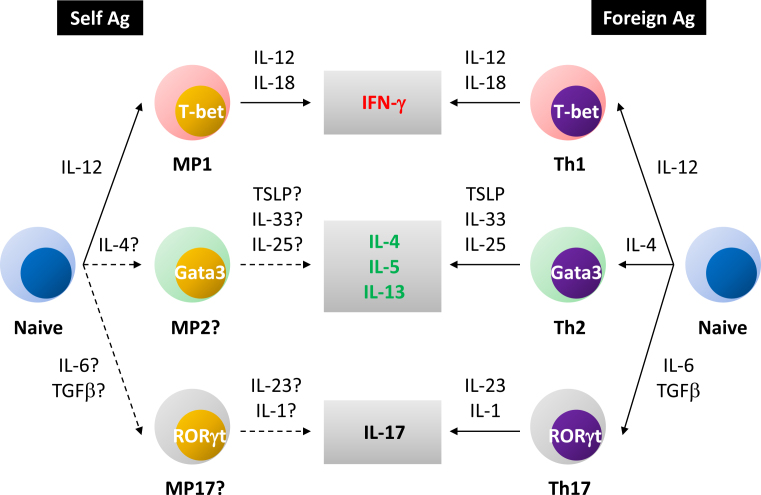
Homeostatic differentiation of MP cells. (Right panel) In conventional helper T cell immune responses, a naïve CD4^+^ T cell specific for a foreign Ag differentiates into Th1, Th2, or Th17 subsets in the influence of the indicated cytokines. These subsets produce different types of effector cytokines. (Left panel) A naïve cell also responds to self-Ag to convert to MP cells. In the presence of IL-12, the latter cells differentiate into T-bet^+^ MP1 at homeostasis and can produce IFN-γ in response to IL-12 and IL-18 in pathogen infection. Presumptive MP2/17 differentiation pathways are also depicted.

At homeostasis, MP CD4^+^ T lymphocytes consist of a dominant T-bet^+^ and a minor T-bet^-^ subpopulations ^(13)^, the former of which we have been referring to as “MP1.” Expectedly, MP cells and especially their MP1 subset can produce IFN-γ when appropriately stimulated ^(13)^. Hence, type 1 differentiation seems to be a unique feature of homeostatic proliferation maintaining self-reactive MP lymphocytes (Figure 2). Additionally, we have found a small fraction of MP cells expressing non-type 1 transcription factors Gata3 and RORγt at low levels ^(12)^. Consistently, a few percent of MP CD4^+^ T lymphocytes can produce cytokines IL-13/17 upon ex vivo stimulation (T.K., unpublished observations). Whether these type 2/17-like subsets represent self-driven “MP2/17” cells remains to be elucidated (Figure 2).

In the case of conventional Th1 differentiation, IL-12 directs naïve T cells to differentiate into a Th1 subset ^(24), (25), (26)^. Recently we have found that homeostatic MP1 differentiation is also significantly promoted by the same cytokine ^(13)^. Bioactive IL-12 p70 is composed of p40 and p35 subunits, and both of these molecules seem to be tonically produced by type 1 DCs (DC1s) in lymphoid tissues at low levels under steady state. Because this IL-12 production is not altered in GF or AF mice, the same response is believed to be driven by some self-derived agonists. Interestingly, this cytokine production, as well as MP1 differentiation, are significantly reduced in mice deficient in *Tlr7* or *Myd88* ^(13)^, suggesting that the TLR7-MyD88 pathway plays a key role in the tonic IL-12 production. In this regard, single-strand RNA of self-origin is known to activate TLR7 ^(57)^. It is thus possible to speculate that the autonomous TLR7 activation tonically stimulates MyD88 signaling in DC1s to drive and/or augment their cytokine production.

**Figure 3.**
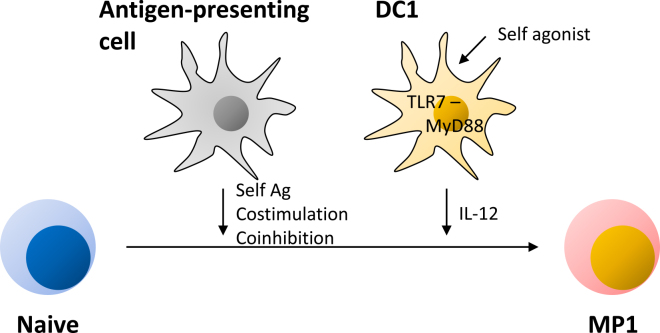
Contribution of different types of antigen-presenting cells to MP1 development. For MP cell development, antigen-presenting cells including DCs are essential to provide naïve precursors with self-Ag/MHC as well as costimulatory/coinhibitory signals. Additionally, DC1s play an inevitable role in homeostatic MP1 differentiation. This DC subset tonically produces IL-12 in response to some self-derived agonists (unidentified) via TLR7-MyD88 signaling pathway in steady state.

Although DC1s play an inevitable role in homeostatic MP1 differentiation, this DC subset does not seem to be essential for homeostatic MP generation itself given that the total MP cell number is unaltered in *Batf3*-deficient mice that specifically lack DC1s ^(13)^. Because MP cell generation requires Ag recognition and because DCs play an important role in this response, two processes, steady state generation of MP cells and their tonic differentiation, are likely to be governed by different types of DC subsets (Figure 3).

Importantly, once MP1 cells are generated, they can exert innate immunological function. Thus, these cells produce IFN-γ in response to high levels of IL-12 in the absence of Ag recognition, as can be seen in NK cell and innate lymphoid cell (ILC) activation, and contribute to host protection from *Toxoplasma* and *Mycobacterium* infection ^(12), (13)^. Moreover, this innate IFN-γ enhances later development of Ag-specific Th1 immune responses, bridging innate and adaptive immunity ^(12)^. Intriguingly, this innate feature of MP CD4^+^ T lymphocytes seems to be shared by CD8^+^ MP (also referred to as virtual memory cells) as well as foreign Ag-specific memory cells. Specifically, these CD8^+^ T lymphocytes produce IFN-γ in response to cytokines to provide bystander protection against *Listeria* infection ^(58), (59), (60), (61), (62)^. Based on these findings, my group and others have proposed that CD4^+^ as well as CD8^+^ MP cells significantly contribute to the lymphocyte-mediated innate immunity provided by NK cells and ILCs, especially at an early phase of pathogen infection before Ag-specific immune responses have fully developed ^(14), (63), (64)^.

Regarding the molecular mechanism of the above innate immune activity of MP cells, it is well known that effector cytokine production of conventional helper T lymphocytes is promoted by various types of environmental cytokines via a combination of STAT and NFκB signaling in vitro ^(65)^. For example, IFN-γ production by Th1 cells is augmented by IL-12 (STAT4 activator) and IL-18 (NFκB activator), whereas Th2 cytokine responses are enhanced by TSLP (STAT5 activator) and IL-33 (NFκB activator). The latter response is further upregulated by IL-25. As for Th17 cells, they produce IL-17 in the influence of IL-23 (STAT3 activator) and IL-1 (NFκB activator). Given the similarities between conventional and homeostatic differentiation, it is suggested that the cytokine-driven activation mechanisms are also operating in MP CD4^+^ T lymphocytes in vivo. Specifically, T-bet^+^ MP1 cells produce IFN-γ in response to IL-12 and IL-18 in the absence of Ag recognition, contributing to host defense against pathogens that induce type 1 immunity. Similarly, hypothetical MP2/17 cells could exhibit such cytokine reactivity to exert innate immune function in type 2/3 infection (Figure 2).

## Clinical Implications

Based on all the above findings on MP cell biology, I propose to deliberately boost MP CD4^+^ T lymphocytes as a novel therapeutic strategy for infectious diseases. Theoretically, this can be achieved by enhancing the generation, proliferation, differentiation, or activation of MP cells. For example, the Flt3 ligand is known to increase the total number of DCs, whereas several types of TLR agonists including TLR7 stimulator imiquimod can further activate DCs, both of which have been proposed as attractive therapeutic agents in the field of HIV research ^(66)^. Because these drugs possibly promote homeostatic proliferation and/or differentiation of MP cells through increment and/or activation of DCs involved in these responses, they might be also useful in combating other infectious diseases. Additionally, stimulation of MP cell costimulatory molecules, including OX40, GITR, and 4-1BB, or blockade of coinhibitory signaling such as CTLA-4 and PD-1, could also be tested ^(55)^.

Among the above strategies, treatment with cytokines seems to be the most promising considering that these molecules can act on MP cells very rapidly. Indeed in mice, exogenous IL-12 treatment has been shown to lead to MP1 differentiation/activation, thereby prolonging survival in *Toxoplasma* infection ^(12)^. By the same token, hypothetical MP2 and MP17 cells could be activated by TSLP/IL-33/IL-25 and IL-23/IL-1, respectively. Because in these procedures MP cells are to be activated independently of foreign Ag specificities, this treatment could target a wide range of pathogens at an early phase of infection, which is in sharp contrast to conventional therapies using antibacterial or antiviral drugs where each agent has a limited range of targets. Such host-directed therapies may be of particular benefit in combating emerging and re-emerging pathogens as well as those with multidrug resistance ^(3)^. Additionally, similar approaches may apply to antitumor treatment.

Because MP cells have TCRs with high affinity to self-Ags, the “MP cell activation therapy” could induce autoimmunity as an adverse effect. In this case, cytokines and/or costimulatory molecules may need to be blocked. In mice, blockade of CD28 and IL-12 signaling has been shown to inhibit proliferation and effector function of MP cells, respectively ^(12)^, which might be useful as the treatment.

The above consideration also leads us to the question of whether or not MP CD4^+^ T lymphocytes are the fundamental cause of an autoimmune disease. As an inevitable consequence of positive selection in the thymus, all T lymphocytes have self-reactivity in the periphery. While this reactivity enables T cells to convert to MP cells possessing innate immune function, it may also generate a risk to respond to self-Ags too strongly. In healthy conditions, such inflammatogenic nature of MP cells is believed to be inhibited by Tregs and coinhibitory signals; nevertheless, under the situation where these mechanisms are disrupted, MP cells may induce an autoimmune disease. If this hypothesis is the case, inhibition of MP cell generation, proliferation, differentiation, or effector function could be a novel therapeutic strategy to treat the disease.

## Article Information

This article is based on the study, which received the Medical Research Encouragement Prize of The Japan Medical Association in 2021.

### Conflicts of Interest

The author declares that there are no conflicts of interest.

### Sources of Funding

This work was supported by the Japan Society for the Promotion of Science, Astellas Foundation for Research on Metabolic Disorders, Bristol-Myers Squibb, Daiichi Sankyo Foundation of Life Science, Kobayashi Foundation, Mochida Memorial Foundation for Medical and Pharmaceutical Research, Life Science Foundation of Japan, Ohyama Health Foundation, Senshin Medical Research Foundation, Takeda Science Foundation, The Cell Science Research Foundation, The Chemo-Sero-Therapeutic Research Institute, The Mitsubishi Foundation, The Sumitomo Foundation, The Uehara Memorial Foundation, and The Waksman Foundation of Japan.

### Acknowledgement

The author gratefully acknowledges A. Sher, J. Sprent, and N. Ishii for their thoughtful discussion.
